# And Now Pennsylvania

**Published:** 1895-11

**Authors:** 


### EDITORIAL.

### AND NOW PENNSYLVANIA.

On another page of this issue will be found a list of the appointments made by Governor Hastings in Pennsylvania to constitute the State Board of Veterinary Medical Examiners. While the law in this State differs from that of New York in that the Governor is not confined in his appointments to any series of names submitted to him by the State Veterinary Association, every effort has been made by those most interested in the success of this measure to have the Governor recognize a member of the faculty of the Veterinary Department of the University of Pennsylvania. In his recognition of Prof. S. J. J. Harger of the above school we feel sure that our chief magistrate has made no mistake. Aside from the high qualifications possessed by the recipient of this appointment, we are confident that by the peculiar experience attained as a professor, instructor, and examiner, he will thus prove the greatest safeguard and restraint upon his fellow-appointees, and thus lessen the danger of graduates of other schools being forced to undergo an unfair test of their ability and worthiness to become practitioners in the Keystone State. We speak advisedly when we say that there has come to our knowledge planned examinations for these applicants in a sister State that would have proved a trying ordeal for any of the graduates to have passed from any of the several colleges of that State or adjacent country, and these proposed questions have come from the sincerest and most earnest supporters of every good veterinary movement, and who are among the strongest advocates of higher education.

